# Supplementary material for: First characterization of the probiotic potential of lactic acid bacteria isolated from Costa Rican pineapple silages
Source: PeerJ. 2021 Nov 30;9:e12437. doi: 10.7717/peerj.12437 (PMC8641478; doi:10.7717/peerj.12437)
Supplement: Supplemental Information 1 [file peerj-09-12437-s001.doc]

| **Table S1 GenBank accession numbers of 16S rRNA gene and phenylalanyl-tRNA synthase gene (*phe*S) sequences from lactic acid bacteria (LAB) isolated from pineapple peel silage.** | | |
| --- | --- | --- |
| **LAB strain** | **GenBank accession number** | |
|  | **16S rRNA** | ***phe*S** |
| *L. paracasei*_6709 | MH753098 | MH752084 |
| *L. paracasei*_6710 | MH753094 | MH752080 |
| *L. paracasei*_6711 | MH753095 | MH752081 |
| *L. paracasei*_6712 | MH753096 | MH752082 |
| *L. paracasei*_6713 | MH753099 | MH752085 |
| *L. paracasei*_6714 | MH753097 | MH752083 |
| *L. paracasei*_6715 | MH753100 | MH752086 |
| *L. fermentum_*6702 | MH753090 | MH752076 |
| *L. fermentum*_6704 | MH753091 | MH752077 |
| *L. parafarraginis_*6717 | MH753092 | MH752078 |
| *L. parafarraginis_*6719 | MH753093 | MH752079 |
| *W. ghanensis_*6706 | MH753101 | MH752087 |
