# Supplementary figures and images for: First characterization of the probiotic potential of lactic acid bacteria isolated from Costa Rican pineapple silages
Source: PeerJ. 2021 Nov 30;9:e12437. doi: 10.7717/peerj.12437 (PMC8641478; doi:10.7717/peerj.12437)

A

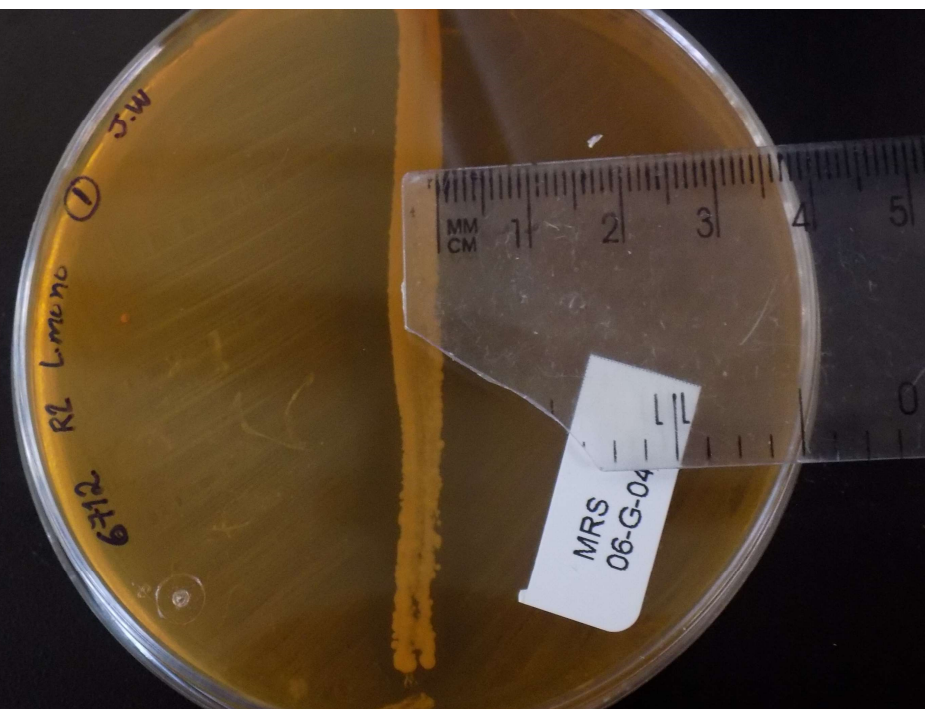

C

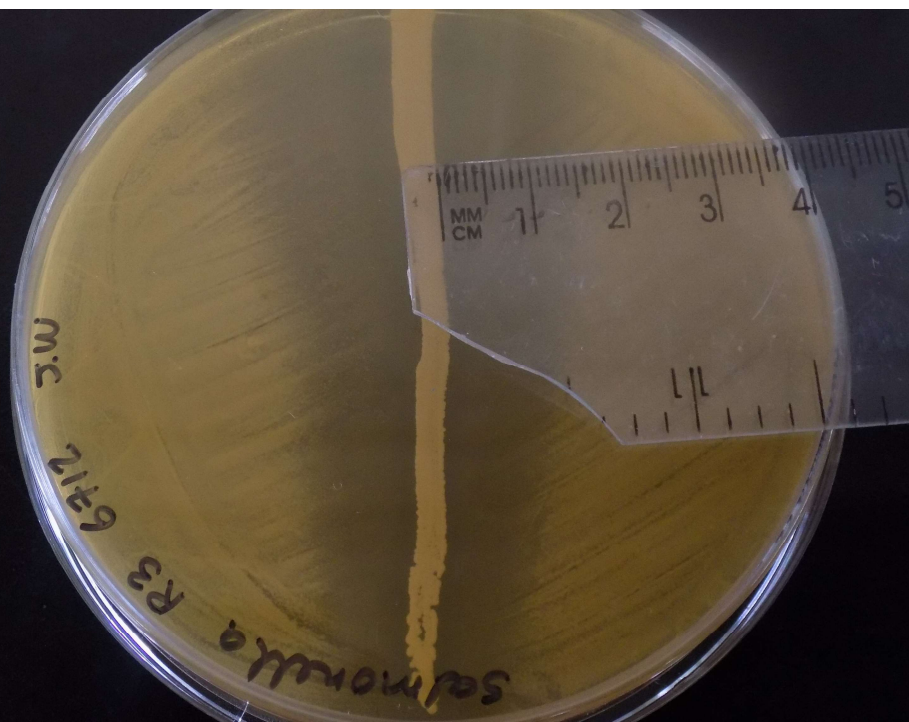

B

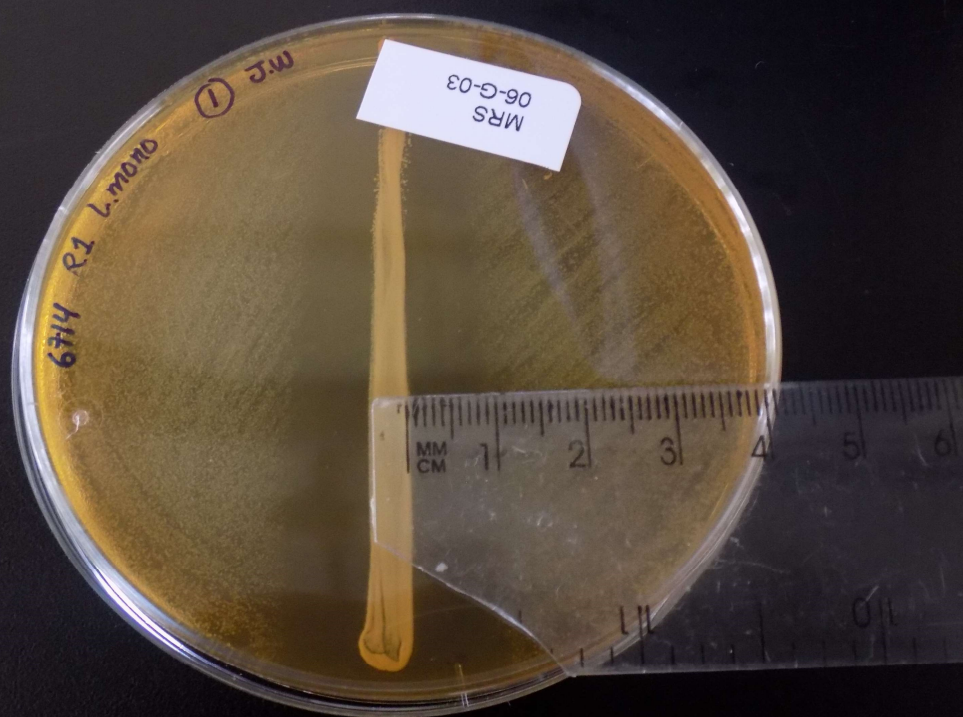

D

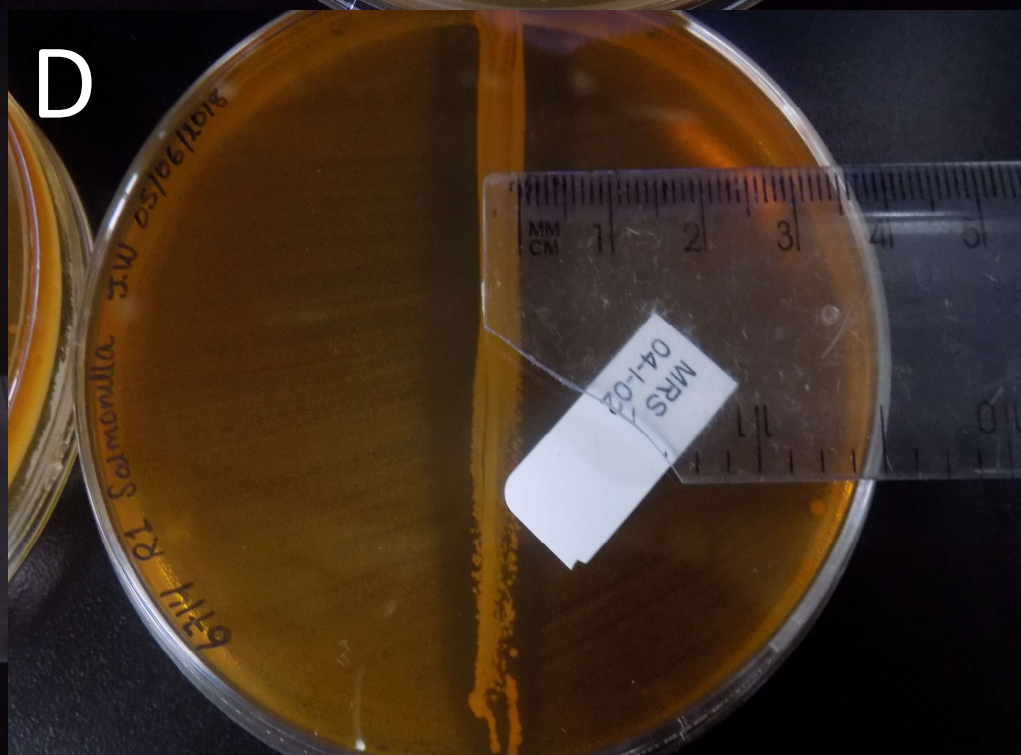

Supplement: Supplemental Information 2 [file peerj-09-12437-s002.pdf]

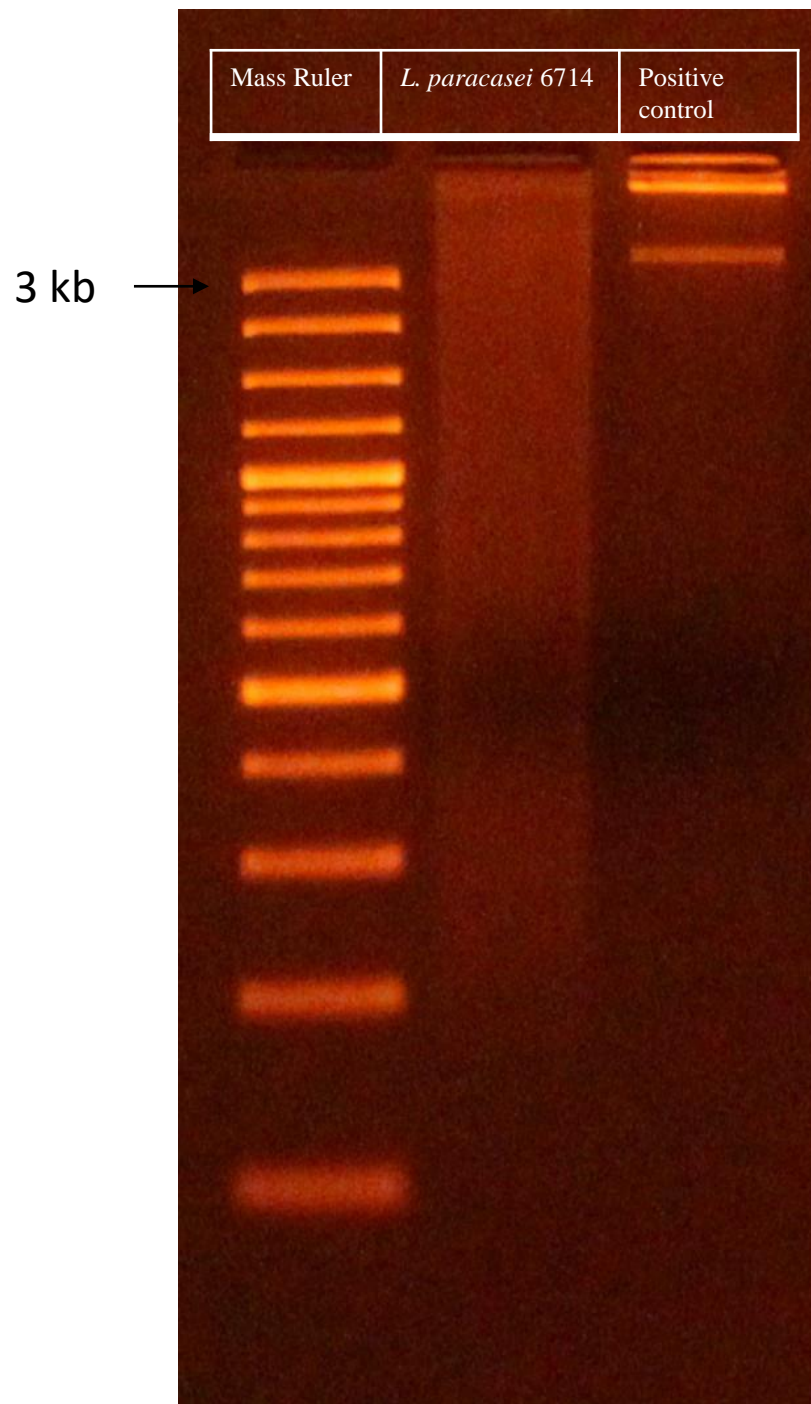

Supplement: Supplemental Information 3 — Gel order:100 bp MassRuler DNA ladder, miniprep of L. paracasei_6714, and miniprep of positive control. [file peerj-09-12437-s003.pdf]
