# Supplementary material for: First characterization of the probiotic potential of lactic acid bacteria isolated from Costa Rican pineapple silages
Source: PeerJ. 2021 Nov 30;9:e12437. doi: 10.7717/peerj.12437 (PMC8641478; doi:10.7717/peerj.12437)
Supplement: Supplemental Information 4 [file peerj-09-12437-s004.doc]

**Lactobacillus casei strain Lc-P6709 16S ribosomal RNA gene, partial sequence**

GenBank: MH753098.1

[GenBank](https://www.ncbi.nlm.nih.gov/nuccore/MH753098.1?report=genbank) [Graphics](https://www.ncbi.nlm.nih.gov/nuccore/MH753098.1?report=graph) [PopSet](https://www.ncbi.nlm.nih.gov/popset?DbFrom=nuccore&Cmd=Link&LinkName=nuccore_popset&IdsFromResult=1450102909)

>MH753098.1 Lactobacillus casei strain Lc-P6709 16S ribosomal RNA gene, partial sequence

GGGTAACCTGCCCTTAAGTGGGGGATAACATTTGGAAACAGATGCTAATACCGCATAGATCCAAGAACCG

CATGGTTCTTGGCTGAAAGATGGCGTAAGCTATCGCTTTTGGATGGACCCGCGGCGTATTAGCTAGTTGG

TGAGGTAATGGCTCACCAAGGCGATGATACGTAGCCGAACTGAGAGGTTGATCGGCCACATTGGGACTGA

GACACGGCCCAAACTCCTACGGGAGGCAGCAGTAGGGAATCTTCCACAATGGACGCAAGTCTGATGGAGC

AACGCCGCGTGAGTGAAGAAGGCTTTCGGGTCGTAAAACTCTGTTGTTGGAGAAGAATGGTCGGCAGAGT

AACTGTTGTCGGCGTGACGGTATCCAACCAGAAAGCCACGGCTAACTACGTGCCAGCAGCCGCGGTAATA

CGTAGGTGGCAAGCGTTATCCGGATTTATTGGGCGTAAAGCGAGCGCAGGCGGTTTTTTAAGTCTGATGT

GAAAGCCCTCGGCTTAACCGAGGAAGCGCATCGGAAACTGGGAAACTTGAGTGCAGAAGAGGACAGTGGA

ACTCCATGTGTAGCGGTGAAATGCGTAGATATATGGAAGAACACCAGTGGCGAAGGCGGCTGTCTGGTCT

GTAACTGACGCTGAGGCTCGAAAGCATGGGTAGCGAACAGGATTAGATACCCTGGTAGTCCATGCCGTAA

ACGATGAATGCTAGGTGTTGGAGGGTTTCCGCCCTTCAGTGCCGCAGCTAACGCATTAAGCATTCCGCCT

GGGGAGTACGACCGCAAGGTTGAAACTCAAAGGAATTGACGGGGGCCCGCACAAGCGGTGGAGCATGTGG

TTTAATTCGAAGCAACGCGAAGAACCTTACCAGGTCTTGACATCTTTTGATCACCTGAGAGATCAGGTTT

CCCCTTCGGGGGCAAAATGACAGGTGGTGCATGGTTGTCGTCAGCTCGTGTCGTGAGATGTTGGGTTAAG

TCCCGCAACGAGCGCAACCCTTATGACTAGTTGCCAGCATTTAGTTGGGCACTCTAGTAAGACTGCCGGT

GACAAACCGGAGGAAGGTGGGGATGACGTCAAATCATCATGCCCCTTATGACCTGGGCTACACACGTGCT

ACAATGGATGGTACAACGAGTTGCGAGACCGCGAGGTCAAGCTAATCTCTTAAAGCCATTCTCAGTTCGG

ACTGTAGGCTGCAACTCGCCTACACGAAGTCGGAATCGCTAGTAATCGCGGATCAGCACGCCGCGGTGAA

TACGTTCCCGGGCCTTGTACACACCGCCCGTCACACCA

<https://www.ncbi.nlm.nih.gov/nuccore/MH753098.1?report=fasta>

# Lactobacillus paracasei strain Lp-P6710 16S ribosomal RNA gene, partial sequence

GenBank: MH753094.1

[GenBank](https://www.ncbi.nlm.nih.gov/nuccore/MH753094.1?report=genbank) [Graphics](https://www.ncbi.nlm.nih.gov/nuccore/MH753094.1?report=graph) [PopSet](https://www.ncbi.nlm.nih.gov/popset?DbFrom=nuccore&Cmd=Link&LinkName=nuccore_popset&IdsFromResult=1450102905)

>MH753094.1 Lactobacillus paracasei strain Lp-P6710 16S ribosomal RNA gene, partial sequence

GGGTAACCTGCCCTTAAGTGGGGGATAACATTTGGAAACAGATGCTAATACCGCATAGATCCAAGAACCG

CATGGTTCTTGGCTGAAAGATGGCGTAAGCTATCGCTTTTGGATGGACCCGCGGCGTATTAGCTAGTTGG

TGAGGTAATGGCTCACCAAGGCGATGATACGTAGCCGAACTGAGAGGTTGATCGGCCACATTGGGACTGA

GACACGGCCCAAACTCCTACGGGAGGCAGCAGTAGGGAATCTTCCACAATGGACGCAAGTCTGATGGAGC

AACGCCGCGTGAGTGAAGAAGGCTTTCGGGTCGTAAAACTCTGTTGTTGGAGAAGAATGGTCGGCAGAGT

AACTGTTGTCGGCGTGACGGTATCCAACCAGAAAGCCACGGCTAACTACGTGCCAGCAGCCGCGGTAATA

CGTAGGTGGCAAGCGTTATCCGGATTTATTGGGCGTAAAGCGAGCGCAGGCGGTTTTTTAAGTCTGATGT

GAAAGCCCTCGGCTTAACCGAGGAAGCGCATCGGAAACTGGGAAACTTGAGTGCAGAAGAGGACAGTGGA

ACTCCATGTGTAGCGGTGAAATGCGTAGATATATGGAAGAACACCAGTGGCGAAGGCGGCTGTCTGGTCT

GTAACTGACGCTGAGGCTCGAAAGCATGGGTAGCGAACAGGATTAGATACCCTGGTAGTCCATGCCGTAA

ACGATGAATGCTAGGTGTTGGAGGGTTTCCGCCCTTCAGTGCCGCAGCTAACGCATTAAGCATTCCGCCT

GGGGAGTACGACCGCAAGGTTGAAACTCAAAGGAATTGACGGGGGCCCGCACAAGCGGTGGAGCATGTGG

TTTAATTCGAAGCAACGCGAAGAACCTTACCAGGTCTTGACATCTTTTGATCACCTGAGAGATCAGGTTT

CCCCTTCGGGGGCAAAATGACAGGTGGTGCATGGTTGTCGTCAGCTCGTGTCGTGAGATGTTGGGTTAAG

TCCCGCAACGAGCGCAACCCTTATGACTAGTTGCCAGCATTTAGTTGGGCACTCTAGTAAGACTGCCGGT

GACAAACCGGAGGAAGGTGGGGATGACGTCAAATCATCATGCCCCTTATGACCTGGGCTACACACGTGCT

ACAATGGATGGTACAACGAGTTGCGAGACCGCGAGGTCAAGCTAATCTCTTAAAGCCATTCTCAGTTCGG

ACTGTAGGCTGCAACTCGCCTACACGAAGTCGGAATCGCTAGTAATCGCGGATCAGCACGCCGCGGTGAA

TACGTTCCCGGGCCTTGTACACACCGCCCGTCACACCA

<https://www.ncbi.nlm.nih.gov/nuccore/MH753094.1?report=fasta>

# Lactobacillus paracasei strain Lp-P6711 16S ribosomal RNA gene, partial sequence

GenBank: MH753095.1

[GenBank](https://www.ncbi.nlm.nih.gov/nuccore/MH753095.1?report=genbank) [Graphics](https://www.ncbi.nlm.nih.gov/nuccore/MH753095.1?report=graph) [PopSet](https://www.ncbi.nlm.nih.gov/popset?DbFrom=nuccore&Cmd=Link&LinkName=nuccore_popset&IdsFromResult=1450102906)

>MH753095.1 Lactobacillus paracasei strain Lp-P6711 16S ribosomal RNA gene, partial sequence

GGGTAACCTGCCCTTAAGTGGGGGATAACATTTGGAAACAGATGCTAATACCGCATAGATCCAAGAACCG

CATGGTTCTTGGCTGAAAGATGGCGTAAGCTATCGCTTTTGGATGGACCCGCGGCGTATTAGCTAGTTGG

TGAGGTAATGGCTCACCAAGGCGATGATACGTAGCCGAACTGAGAGGTTGATCGGCCACATTGGGACTGA

GGCACGGCCCAAATTCCTACGGGAGGCAGCAGTAGGGAATCTTCCACAATGGACGCAAGTCTGATGGAGC

AACGCCGCGTGAGTGAAGAAGGCTTTCGGGTCGTAAAACTCTGTTGTTGGAGAAGAATGGTCGGCAGAGT

AACTGTTGTCGGCGTGACGGTATCCAACCAGAAAGCCACGGCTAACTACGTGCCAGCAGCCGCGGTAATA

CGTAGGTGGCAAGCGTTATCCGGATTTATTGGGCGTAAAGCGAGCGCAGGCGGTTTTTTAAGTCTGATGT

GAAAGCCCTCGGCTTAACCGAGGAAGCGCATCGGAAACTGGGAAACTTGAGTGCAGAAGAGGACAGTGGA

ACTCCATGTGTAGCGGTGAAATGCGTAGATATATGGAAGAACACCAGTGGCGAAGGCGGCTGTCTGGTCT

GTAACTGACGCTGAGGCTCGAAAGCATGGGTAGCGAACAGGATTAGATACCCTGGTAGTCCATGCCGTAA

ACGATGAATGCTAGGTGTTGGAGGGTTTCCGCCCTTCAGTGCCGCAGCTAACGCATTAAGCATTCCGCCT

GGGGAGTACGACCGCAAGGTTGAAACTCAAAGGAATTGACGGGGGCCCGCACAAGCGGTGGAGCATGTGG

TTTAATTCGAAGCAACGCGAAGAACCTTACCAGGTCTTGACATCTTTTGATCACCTGAGAGATCAGGTTT

CCCCTTCGGGGGCAAAATGACAGGTGGTGCATGGTTGTCGTCAGCTCGTGTCGTGAGATGTTGGGTTAAG

TCCCGCAACGAGCGCAACCCTTATGACTAGTTGCCAGCATTTAGTTGGGCACTCTAGTAAGACTGCCGGT

GACAAACCGGAGGAAGGTGGGGATGACGTCAAATCATCATGCCCCTTATGACCTGGGCTACACACGTGCT

ACAATGGATGGTACAACGAGTTGCGAGACCGCGAGGTCAAGCTAATCTCTTAAAGCCATTCTCAGTTCGG

ACTGTAGGCTGCAACTCGCCTACACGAAGTCGGAATCGCTAGTAATCGCGGATCAGCACGCCGCGGTGAA

TACGTTCCCGGGCCTTGTACACACCGCCCGTCACACCA

<https://www.ncbi.nlm.nih.gov/nuccore/MH753095.1?report=fasta>

# Lactobacillus paracasei strain Lp-P6712 16S ribosomal RNA gene, partial sequence

GenBank: MH753096.1

[GenBank](https://www.ncbi.nlm.nih.gov/nuccore/MH753096.1?report=genbank) [Graphics](https://www.ncbi.nlm.nih.gov/nuccore/MH753096.1?report=graph) [PopSet](https://www.ncbi.nlm.nih.gov/popset?DbFrom=nuccore&Cmd=Link&LinkName=nuccore_popset&IdsFromResult=1450102907)

>MH753096.1 Lactobacillus paracasei strain Lp-P6712 16S ribosomal RNA gene, partial sequence

GGGTAACCTGCCCTTAAGTGGGGGATAACATTTGGAAACAGATGCTAATACCGCATAGATCCAAGAACCG

CATGGTTCTTGGCTGAAAGATGGCGTAAGCTATCGCTTTTGGATGGACCCGCGGCGTATTAGCTAGTTGG

TGAGGTAATGGCTCACCAAGGCGATGATACGTAGCCGAACTGAGAGGTTGATCGGCCACATTGGGACTGA

GACACGGCCCAAACTCCTACGGGAGGCAGCAGTAGGGAATCTTCCACAATGGACGCAAGTCTGATGGAGC

AACGCCGCGTGAGTGAAGAAGGCTTTCGGGTCGTAAAACTCTGTTGTTGGAGAAGAATGGTCGGCAGAGT

AACTGTTGTCGGCGTGACGGTATCCAACCAGAAAGCCACGGCTAACTACGTGCCAGCAGCCGCGGTAATA

CGTAGGTGGCAAGCGTTATCCGGATTTATTGGGCGTAAAGCGAGCGCAGGCGGTTTTTTAAGTCTGATGT

GAAAGCCCTCGGCTTAACCGAGGAAGCGCATCGGAAACTGGGAAACTTGAGTGCAGAAGAGGACAGTGGA

ACTCCATGTGTAGCGGTGAAATGCGTAGATATATGGAAGAACACCAGTGGCGAAGGCGGCTGTCTGGTCT

GTAACTGACGCTGAGGCTCGAAAGCATGGGTAGCGAACAGGATTAGATACCCTGGTAGTCCATGCCGTAA

ACGATGAATGCTAGGTGTTGGAGGGTTTCCGCCCTTCAGTGCCGCAGCTAACGCATTAAGCATTCCGCCT

GGGGAGTACGACCGCAAGGTTGAAACTCAAAGGAATTGACGGGGGCCCGCACAAGCGGTGGAGCATGTGG

TTTAATTCGAAGCAACGCGAAGAACCTTACCAGGTCTTGACATCTTTTGATCACCTGAGAGATCAGGTTT

CCCCTTCGGGGGCAAAATGACAGGTGGTGCATGGTTGTCGTCAGCTCGTGTCGTGAGATGTTGGGTTAAG

TCCCGCAACGAGCGCAACCCTTATGACTAGTTGCCAGCATTTAGTTGGGCACTCTAGTAAGACTGCCGGT

GACAAACCGGAGGAAGGTGGGGATGACGTCAAATCATCATGCCCCTTATGACCTGGGCTACACACGTGCT

ACAATGGATGGTACAACGAGTTGCGAGACCGCGAGGTCAAGCTAATCTCTTAAAGCCATTCTCAGTTCGG

ACTGTAGGCTGCAACTCGCCTACACGAAGTCGGAATCGCTAGTAATCGCGGATCAGCACGCCGCGGTGAA

TACGTTCCCGGGCCTTGTACACACCGCCCGTCACACCA

<https://www.ncbi.nlm.nih.gov/nuccore/MH753096.1?report=fasta>

# Lactobacillus casei strain Lc-P6713 16S ribosomal RNA gene, partial sequence

GenBank: MH753099.1

[GenBank](https://www.ncbi.nlm.nih.gov/nuccore/MH753099.1?report=genbank) [Graphics](https://www.ncbi.nlm.nih.gov/nuccore/MH753099.1?report=graph) [PopSet](https://www.ncbi.nlm.nih.gov/popset?DbFrom=nuccore&Cmd=Link&LinkName=nuccore_popset&IdsFromResult=1450102910)

>MH753099.1 Lactobacillus casei strain Lc-P6713 16S ribosomal RNA gene, partial sequence

GGGTAACCTGCCCTTAAGTGGGGGATAACATTTGGAAACAGATGCTAATACCGCATAGATCCAAGAACCG

CATGGTTCTTGGCTGAAAGATGGCGTAAGCTATCGCTTTTGGATGGACCCGCGGCGTATTAGCTAGTTGG

TGAGGTAATGGCTCACCAAGGCGATGATACGTAGCCGAACTGAGAGGTTGATCGGCCACATTGGGACTGA

GACACGGCCCAAACTCCTACGGGAGGCAGCAGTAGGGAATCTTCCACAATGGACGCAAGTCTGATGGAGC

AACGCCGCGTGAGTGAAGAAGGCTTTCGGGTCGTAAAACTCTGTTGTTGGAGAAGAATGGTCGGCAGAGT

AACTGTTGTCGGCGTGACGGTATCCAACCAGAAAGCCACGGCTAACTACGTGCCAGCAGCCGCGGTAATA

CGTAGGTGGCAAGCGTTATCCGGATTTATTGGGCGTAAAGCGAGCGCAGGCGGTTTTTTAAGTCTGATGT

GAAAGCCCTCGGCTTAACCGAGGAAGCGCATCGGAAACTGGGAAACTTGAGTGCAGAAGAGGACAGTGGA

ACTCCATGTGTAGCGGTGAAATGCGTAGATATATGGAAGAACACCAGTGGCGAAGGCGGCTGTCTGGTCT

GTAACTGACGCTGAGGCTCGAAAGCATGGGTAGCGAACAGGATTAGATACCCTGGTAGTCCATGCCGTAA

ACGATGAATGCTAGGTGTTGGAGGGTTTCCGCCCTTCAGTGCCGCAGCTAACGCATTAAGCATTCCGCCT

GGGGAGTACGACCGCAAGGTTGAAACTCAAAGGAATTGACGGGGGCCCGCACAAGCGGTGGAGCATGTGG

TTTAATTCGAAGCAACGCGAAGAACCTTACCAGGTCTTGACATCTTTTGATCACCTGAGAGATCAGGTTT

CCCCTTCGGGGGCAAAATGACAGGTGGTGCATGGTTGTCGTCAGCTCGTGTCGTGAGATGTTGGGTTAAG

TCCCGCAACGAGCGCAACCCTTATGACTAGTTGCCAGCATTTAGTTGGGCACTCTAGTAAGACTGCCGGT

GACAAACCGGAGGAAGGTGGGGATGACGTCAAATCATCATGCCCCTTATGACCTGGGCTACACACGTGCT

ACAATGGATGGTACAACGAGTTGCGAGACCGCGAGGTCAAGCTAATCTCTTAAAGCCATTCTCAGTTCGG

ACTGTAGGCTGCAACTCGCCTACACGAAGTCGGAATCGCTAGTAATCGCGGATCAGCACGCCGCGGTGAA

TACGTTCCCGGGCCTTGTACACACCGCCCGTCACACCA

<https://www.ncbi.nlm.nih.gov/nuccore/MH753099.1?report=fasta>

# Lactobacillus paracasei strain Lp-P6714 16S ribosomal RNA gene, partial sequence

GenBank: MH753097.1

[GenBank](https://www.ncbi.nlm.nih.gov/nuccore/MH753097.1?report=genbank) [Graphics](https://www.ncbi.nlm.nih.gov/nuccore/MH753097.1?report=graph) [PopSet](https://www.ncbi.nlm.nih.gov/popset?DbFrom=nuccore&Cmd=Link&LinkName=nuccore_popset&IdsFromResult=1450102908)

>MH753097.1 Lactobacillus paracasei strain Lp-P6714 16S ribosomal RNA gene, partial sequence

GGGTAACCTGCCCTTAAGTGGGGGATAACATTTGGAAACAGATGCTAATACCGCATAGATCCAAGAACCG

CATGGTTCTTGGCTGAAAGATGGCGTAAGCTATCGCTTTTGGATGGACCCGCGGCGTATTAGCTAGTTGG

TGAGGTAATGGCTCACCAAGGCGATGATACGTAGCCGAACTGAGAGGTTGATCGGCCACATTGGGACTGA

GACACGGCCCAAACTCCTACGGGAGGCAGCAGTAGGGAATCTTCCACAATGGACGCAAGTCTGATGGAGC

AACGCCGCGTGAGTGAAGAAGGCTTTCGGGTCGTAAAACTCTGTTGTTGGAGAAGAATGGTCGGCAGAGT

AACTGTTGTCGGCGTGACGGTATCCAACCAGAAAGCCACGGCTAACTACGTGCCAGCAGCCGCGGTAATA

CGTAGGTGGCAAGCGTTATCCGGATTTATTGGGCGTAAAGCGAGCGCAGGCGGTTTTTTAAGTCTGATGT

GAAAGCCCTCGGCTTAACCGAGGAAGCGCATCGGAAACTGGGAAACTTGAGTGCAGAAGAGGACAGTGGA

ACTCCATGTGTAGCGGTGAAATGCGTAGATATATGGAAGAACACCAGTGGCGAAGGCGGCTGTCTGGTCT

GTAACTGACGCTGAGGCTCGAAAGCATGGGTAGCGAACAGGATTAGATACCCTGGTAGTCCATGCCGTAA

ACGATGAATGCTAGGTGTTGGAGGGTTTCCGCCCTTCAGTGCCGCAGCTAACGCATTAAGCATTCCGCCT

GGGGAGTACGACCGCAAGGTTGAAACTCAAAGGAATTGACGGGGGCCCGCACAAGCGGTGGAGCATGTGG

TTTAATTCGAAGCAACGCGAAGAACCTTACCAGGTCTTGACATCTTTTGATCACCTGAGAGATCAGGTTT

CCCCTTCGGGGGCAAAATGACAGGTGGTGCATGGTTGTCGTCAGCTCGTGTCGTGAGATGTTGGGTTAAG

TCCCGCAACGAGCGCAACCCTTATGACTAGTTGCCAGCATTTAGTTGGGCACTCTAGTAAGACTGCCGGT

GACAAACCGGAGGAAGGTGGGGATGACGTCAAATCATCATGCCCCTTATGACCTGGGCTACACACGTGCT

ACAATGGATGGTACAACGAGTTGCGAGACCGCGAGGTCAAGCTAATCTCTTAAAGCCATTCTCAGTTCGG

ACTGTAGGCTGCAACTCGCCTACACGAAGTCGGAATCGCTAGTAATCGCGGATCAGCACGCCGCGGTGAA

TACGTTCCCGGGCCTTGTACACACCGCCCGTCACACCA

<https://www.ncbi.nlm.nih.gov/nuccore/MH753097.1?report=fasta>

# Lactobacillus casei strain Lc-P6715 16S ribosomal RNA gene, partial sequence

GenBank: MH753100.1

[GenBank](https://www.ncbi.nlm.nih.gov/nuccore/MH753100.1?report=genbank) [Graphics](https://www.ncbi.nlm.nih.gov/nuccore/MH753100.1?report=graph) [PopSet](https://www.ncbi.nlm.nih.gov/popset?DbFrom=nuccore&Cmd=Link&LinkName=nuccore_popset&IdsFromResult=1450102911)

>MH753100.1 Lactobacillus casei strain Lc-P6715 16S ribosomal RNA gene, partial sequence

GGGTAACCTGCCCTTAAGTGGGGGATAACATTTGGAAACAGATGCTAATACCGCATAGATCCAAGAACCG

CATGTTTTTTGGCTGAAAGATGGCGTAAGCTATCGCTTTTGGATGGACCCGCGGCGTATTAGCTAGTTGG

TGAGGTAATGGCTCACCAAGGCGATGATACGTAGCCGAACTGAGAGGTTGATCGGCCACATTGGGACTGA

GACACGGCCCAAATTCCTACGGGAGGCAGCAGTAGGGAATCTTCCACAATGGACGCAAGTCTGATGGAGC

AACGCCGCGTGAGTGAAGAAGGCTTTCGGGTCGTAAAACTCTGTTGTTGGAGAAGAATGGTCGGCAGAGT

AACTGTTGTCGGCGTGACGGTATCCAACCAGAAAGCCACGGCTAACTACGTGCCAGCAGCCGCGGTAATA

CGTAGGTGGCAAGCGTTATCCGGATTTATTGGGCGTAAAGCGAGCGCAGGCGGTTTTTTAAGTCTGATGT

GAAAGCCCTCGGCTTAACCGAGGAAGCGCATCGGAAACTGGGAAACTTGAGTGCAGAAGAGGACAGTGGA

ACTCCATGTGTAGCGGTGAAATGCGTAGATATATGGAAGAACACCAGTGGCGAAGGCGGCTGTCTGGTCT

GTAACTGACGCTGAGGCTCGAAAGCATGGGTAGCGAACAGGATTAGATACCCTGGTAGTCCATGCCGTAA

ACGATGAATGCTAGGTGTTGGAGGGTTTCCGCCCTTCAGTGCCGCAGCTAACGCATTAAGCATTCCGCCT

GGGGAGTACGACCGCAAGGTTGAAACTCAAAGGAATTGACGGGGGCCCGCACAAGCGGTGGAGCATGTGG

TTTAATTCGAAGCAACGCGAAGAACCTTACCAGGTCTTGACATCTTTTGATCACCTGAGAGATCAGGTTT

CCCCTTCGGGGGCAAAATGACAGGTGGTGCATGGTTGTCGTCAGCTCGTGTCGTGAGATGTTGGGTTAAG

TCCCGCAACGAGCGCAACCCTTATGACTAGTTGCCAGCATTTAGTTGGGCACTCTAGTAAGACTGCCGGT

GACAAACCGGAGGAAGGTGGGGATGACGTCAAATCATCATGCCCCTTATGACCTGGGCTACACACGTGCT

ACAATGGATGGTACAACGAGTTGCGAGACCGCGAGGTCAAGCTAATCTCTTAAAGCCATTCTCAGTTCGG

ACTGTAGGCTGCAACTCGCCTACACGAAGTCGGAATCGCTAGTAATCGCGGATCAGCACGCCGCGGTGAA

TACGTTCCCGGGCCTTGTACACACCGCCCGTCACACCA

<https://www.ncbi.nlm.nih.gov/nuccore/MH753100.1?report=fasta>

# Lactobacillus fermentum strain Lf-P6702 16S ribosomal RNA gene, partial sequence

GenBank: MH753090.1

[GenBank](https://www.ncbi.nlm.nih.gov/nuccore/MH753090.1?report=genbank) [Graphics](https://www.ncbi.nlm.nih.gov/nuccore/MH753090.1?report=graph) [PopSet](https://www.ncbi.nlm.nih.gov/popset?DbFrom=nuccore&Cmd=Link&LinkName=nuccore_popset&IdsFromResult=1450102901)

>MH753090.1 Lactobacillus fermentum strain Lf-P6702 16S ribosomal RNA gene, partial sequence

AGGTAACCTGCCCAGAAGCGGGGGACAACATTTGGAAACAGATGCTAATACCGCATAACAACGTTGTTCG

CATGAACAACGCTTAAAAGATGGCTTCTCGCTATCACTTCTGGATGGACCTGCGGTGCATTAGCTTGTTG

GTGGGGTAACGGCCTACCAAGGCGATGATGCATAGCCGAGTTGAGAGACTGATCGGCCACAATGGGACTG

AGACACGGCCCATACTCCTACGGGAGGCAGCAGTAGGGAATCTTCCACAATGGGCGCAAGCCTGATGGAG

CAACACCGCGTGAGTGAAGAAGGGTTTCGGCTCGTAAAGCTCTGTTGTTAAAGAAGAACACGTATGAGAG

TAACTGTTCATACGTTGACGGTATTTAACCAGAAAGTCACGGCTAACTACGTGCCAGCAGCCGCGGTAAT

ACGTAGGTGGCAAGCGTTATCCGGATTTATTGGGCGTAAAGAGAGTGCAGGCGGTTTTCTAAGTCTGATG

TGAAAGCCTTCGGCTTAACCGGAGAAGTGCATCGGAAACTGGATAACTTGAGTGCAGAAGAGGGTAGTGG

AACTCCATGTGTAGCGGTGGAATGCGTAGATATATGGAAGAACACCAGTGGCGAAGGCGGCTACCTGGTC

TGCAACTGACGCTGAGACTCGAAAGCATGGGTAGCGAACAGGATTAGATACCCTGGTAGTCCATGCCGTA

AACGATGAGTGCTAGGTGTTGGAGGGTTTCCGCCCTTCAGTGCCGGAGCTAACGCATTAAGCACTCCGCC

TGGGGAGTACGACCGCAAGGTTGAAACTCAAAGGAATTGACGGGGGCCCGCACAAGCGGTGGAGCATGTG

GTTTAATTCGAAGCTACGCGAAGAACCTTACCAGGTCTTGACATCTTGCGCCAACCCTAGAGATAGGGCG

TTTCCTTCGGGAACGCAATGACAGGTGGTGCATGGTCGTCGTCAGCTCGTGTCGTGAGATGTTGGGTTAA

GTCCCGCAACGAGCGCAACCCTTGTTACTAGTTGCCAGCATTAAGTTGGGCACTCTAGTGAGACTGCCGG

TGACAAACCGGAGGAAGGTGGGGACGACGTCAGATCATCATGCCCCTTATGACCTGGGCTACACACGTGC

TACAATGGACGGTACAACGAGTCGCGAACTCGCGAGGGCAAGCAAATCTCTTAAAACCGTTCTCAGTTCG

GACTGCAGGCTGCAACTCGCCTGCACGAAGTCGGAATCGCTAGTAATCGCGGATCAGCATGCCGCGGTGA

ATACGTTCCCGGGCCTTGTACACACCGCCCGTCACACCA

<https://www.ncbi.nlm.nih.gov/nuccore/MH753090.1?report=fasta>

# Lactobacillus fermentum strain Lf-P6704 16S ribosomal RNA gene, partial sequence

GenBank: MH753091.1

[GenBank](https://www.ncbi.nlm.nih.gov/nuccore/MH753091.1?report=genbank) [Graphics](https://www.ncbi.nlm.nih.gov/nuccore/MH753091.1?report=graph) [PopSet](https://www.ncbi.nlm.nih.gov/popset?DbFrom=nuccore&Cmd=Link&LinkName=nuccore_popset&IdsFromResult=1450102902)

>MH753091.1 Lactobacillus fermentum strain Lf-P6704 16S ribosomal RNA gene, partial sequence

AGGTAACCTGCCCAGAAGCGGGGGACAACATTTGGAAACAGATGCTAATACCGCATAACAACGTTGTTCG

CATGAACAACGCTTAAAAGATGGCTTCTCGCTATCACTTCTGGATGGACCTGCGGTGCATTAGCTTGTTG

GTGGGGTAACGGCCTACCAAGGCGATGATGCATAGCCGAGTTGAGAGACTGATCGGCCACAATGGGACTG

AGACACGGCCCATACTCCTACGGGAGGCAGCAGTAGGGAATCTTCCACAATGGGCGCAAGCCTGATGGAG

CAACACCGCGTGAGTGAAGAAGGGTTTCGGCTCGTAAAGCTCTGTTGTTAAAGAAGAACACGTATGAGAG

TAACTGTTCATACGTTGACGGTATTTAACCAGAAAGTCACGGCTAACTACGTGCCAGCAGCCGCGGTAAT

ACGTAGGTGGCAAGCGTTATCCGGATTTATTGGGCGTAAAGAGAGTGCAGGCGGTTTTCTAAGTCTGATG

TGAAAGCCTTCGGCTTAACCGGAGAAGTGCATCGGAAACTGGATAACTTGAGTGCAGAAGAGGGTAGTGG

AACTCCATGTGTAGCGGTGGAATGCGTAGATATATGGAAGAACACCAGTGGCGAAGGCGGCTACCTGGTC

TGCAACTGACGCTGAGACTCGAAAGCATGGGTAGCGAACAGGATTAGATACCCTGGTAGTCCATGCCGTA

AACGATGAGTGCTAGGTGTTGGAGGGTTTCCGCCCTTCAGTGCCGGAGCTAACGCATTAAGCACTCCGCC

TGGGGAGTACGACCGCAAGGTTGAAACTCAAAGGAATTGACGGGGGCCCGCACAAGCGGTGGAGCATGTG

GTTTAATTCGAAGCTACGCGAAGAACCTTACCAGGTCTTGACATCTTGCGCCAACCCTAGAGATAGGGCG

TTTCCTTCGGGAACGCAATGACAGGTGGTGCATGGTCGTCGTCAGCTCGTGTCGTGAGATGTTGGGTTAA

GTCCCGCAACGAGCGCAACCCTTGTTACTAGTTGCCAGCATTAAGTTGGGCACTCTAGTGAGACTGCCGG

TGACAAACCGGAGGAAGGTGGGGACGACGTCAGATCATCATGCCCCTTATGACCTGGGCTACACACGTGC

TACAATGGACGGTACAACGAGTCGCGAACTCGCGAGGGCAAGCAAATCTCTTAAAACCGTTCTCAGTTCG

GACTGCAGGCTGCAACTCGCCTGCACGAAGTCGGAATCGCTAGTAATCGCGGATCAGCATGCCGCGGTGA

ATACGTTCCCGGGCCTTGTACACACCGCCCGTCACACCA

<https://www.ncbi.nlm.nih.gov/nuccore/MH753091.1?report=fasta>

# Lactobacillus parafarraginis strain Lp-P6717 16S ribosomal RNA gene, partial sequence

GenBank: MH753092.1

[GenBank](https://www.ncbi.nlm.nih.gov/nuccore/MH753092.1?report=genbank) [Graphics](https://www.ncbi.nlm.nih.gov/nuccore/MH753092.1?report=graph) [PopSet](https://www.ncbi.nlm.nih.gov/popset?DbFrom=nuccore&Cmd=Link&LinkName=nuccore_popset&IdsFromResult=1450102903)

>MH753092.1 Lactobacillus parafarraginis strain Lp-P6717 16S ribosomal RNA gene, partial sequence

GGGTAACCTGCCCTGAAGTGGGGGATAACACTTGGAAACAGGTGCTAATACCGCATAACAACGAAAACCA

CATGGTTTTCGTTTGAAAGATGGCTTCGGCTGTCACTTTTGGATGGACCCGCGGCGTATTAGCTTGTTGG

TGAGGTAACGGCTCACCAAGGCCATGATACGTAGCCGACCTGAGAGGGTAATCGGCCACATTGGGACTGA

GACACGGCCCAAACTCCTACGGGAGGCAGCAGTAGGGAATCTTCCACAATGGACGAAAGTCTGATGGAGC

AACGCCGCGTGAGTGATGAAGGGTTTCGACTCGTAAAACTCTGTTGTTGGAGAAGAACAGGTGATAGAGT

AACTGTTATCATCTTGACGGTATCCAACCAGAAAGCCACGGCTAACTACGTGCCAGCAGCCGCGGTAATA

CGTAGGTGGCAAGCGTTGTCCGGATTTATTGGGCGTAAAGCGAGCGCAGGCGGTTTTTTAGGTCTGATGT

GAAAGCCTTCGGCTTAACCGGAGAAGGGCATCGGAAACCGGGAGACTTGAGTGCAGAAGAGGACAGTGGA

ACTCCATGTGTAGCGGTGAAATGCGTAGATATATGGAAGAACACCAGTGGCGAAGGCGGCTGTCTGGTCT

GTAACTGACGCTGAGGCTCGAAAGCATGGGTAGCGAACAGGATTAGATACCCTGGTAGTCCATGCCGTAA

ACGATGAGTGCTAAGTGTTGGAGGGTTTCCGCCCTTCAGTGCTGCAGCTAACGCATTAAGCACTCCGCCT

GGGGAGTACGACCGCAAGGTTGAAACTCAAAGGAATTGACGGGGGCCCGCACAAGCGGTGGAGCATGTGG

TTTAATTCGATGCTACGCGAAGAACCTTACCAGGTCTTGACATCTTCTGCTAACCTAAGAGATTAGGCGT

TCCCTTCGGGGACGGAATGACAGGTGGTGCATGGTTGTCGTCAGCTCGTGTCGTGAGATGTTGGGTTAAG

TCCCGCAACGAGCGCAACCCTTATTGTCAGTTGCCAGCATTTAGTTGGGCACTCTGGCGAGACTGCCGGT

GACAAACCGGAGGAAGGTGGGGATGACGTCAAATCATCATGCCCCTTATGACCTGGGCTACACACGTGCT

ACAATGGACGGTACAACGAGTCGCGAAACCGCGAGGTCAAGCTAATCTCTTAAAGCCGTTCTCAGTTCGG

ATTGCAGGCTGCAACTCGCCTGCATGAAGTTGGAATCGCTAGTAATCGTGGATCAGCATGCCACGGTGAA

TACGTTCCCGGGCCTTGTACACACCGCCCGTCACACCA

<https://www.ncbi.nlm.nih.gov/nuccore/MH753092.1?report=fasta>

# Lactobacillus parafarraginis strain Lp-P6719 16S ribosomal RNA gene, partial sequence

GenBank: MH753093.1

[GenBank](https://www.ncbi.nlm.nih.gov/nuccore/MH753093.1?report=genbank) [Graphics](https://www.ncbi.nlm.nih.gov/nuccore/MH753093.1?report=graph) [PopSet](https://www.ncbi.nlm.nih.gov/popset?DbFrom=nuccore&Cmd=Link&LinkName=nuccore_popset&IdsFromResult=1450102904)

>MH753093.1 Lactobacillus parafarraginis strain Lp-P6719 16S ribosomal RNA gene, partial sequence

GGGTAACCTGCCCTGAAGTGGGGGATAACACTTGGAAACAGGTGCTAATACCGCATAACAACGAAAACCA

CATGGTTTTCGTTTGAAAGATGGCTTCGGCTGTCACTTTTGGATGGACCCGCGGCGTATTAGCTTGTTGG

TGAGGTAACGGCTCACCAAGGCCATGATACGTAGCCGACCTGAGAGGGTAATCGGCCACATTGGGACTGA

GACACGGCCCAAACTCCTACGGGAGGCAGCAGTAGGGAATCTTCCACAATGGACGAAAGTCTGATGGAGC

AACGCCGCGTGAGTGATGAAGGGTTTCGACTCGTAAAACTCTGTTGTTGGAGAAGAACAGGTGATAGAGT

AACTGTTATCATCTTGACGGTATCCAACCAGAAAGCCACGGCTAACTACGTGCCAGCAGCCGCGGTAATA

CGTAGGTGGCAAGCGTTGTCCGGATTTATTGGGCGTAAAGCGAGCGCAGGCGGTTTTTTAGGTCTGATGT

GAAAGCCTTCGGCTTAACCGGAGAAGGGCATCGGAAACCGGGAGACTTGAGTGCAGAAGAGGACAGTGGA

ACTCCATGTGTAGCGGTGAAATGCGTAGATATATGGAAGAACACCAGTGGCGAAGGCGGCTGTCTGGTCT

GTAACTGACGCTGAGGCTCGAAAGCATGGGTAGCGAACAGGATTAGATACCCTGGTAGTCCATGCCGTAA

ACGATGAGTGCTAAGTGTTGGAGGGTTTCCGCCCTTCAGTGCTGCAGCTAACGCATTAAGCACTCCGCCT

GGGGAGTACGACCGCAAGGTTGAAACTCAAAGGAATTGACGGGGGCCCGCACAAGCGGTGGAGCATGTGG

TTTAATTCGATGCTACGCGAAGAACCTTACCAGGTCTTGACATCTTCTGCTAACCTAAGAGATTAGGCGT

TCCCTTCGGGGACGGAATGACAGGTGGTGCATGGTTGTCGTCAGCTCGTGTCGTGAGATGTTGGGTTAAG

TCCCGCAACGAGCGCAACCCTTATTGTCAGTTGCCAGCATTTAGTTGGGCACTCTGGCGAGACTGCCGGT

GACAAACCGGAGGAAGGTGGGGATGACGTCAAATCATCATGCCCCTTATGACCTGGGCTACACACGTGCT

ACAATGGACGGTACAACGAGTCGCGAAACCGCGAGGTCAAGCTAATCTCTTAAAGCCGTTCTCAGTTCGG

ATTGCAGGCTGCAACTCGCCTGCATGAAGTTGGAATCGCTAGTAATCGTGGATCAGCATGCCACGGTGAA

TACGTTCCCGGGCCTTGTACACACCGCCCGTCACACCA

<https://www.ncbi.nlm.nih.gov/nuccore/MH753093.1?report=fasta>

# Weissella ghanensis strain Wg-P6706 16S ribosomal RNA gene, partial sequence

GenBank: MH753101.1

[GenBank](https://www.ncbi.nlm.nih.gov/nuccore/MH753101.1?report=genbank) [Graphics](https://www.ncbi.nlm.nih.gov/nuccore/MH753101.1?report=graph) [PopSet](https://www.ncbi.nlm.nih.gov/popset?DbFrom=nuccore&Cmd=Link&LinkName=nuccore_popset&IdsFromResult=1450102912)

>MH753101.1 Weissella ghanensis strain Wg-P6706 16S ribosomal RNA gene, partial sequence

GGGAAACCTACCTCTTAGCAGGGGATAACATTTGGAAACAGATGCTAATACCGTATAATAACTAGAACCG

CATGGTTCTAGTTTAAAAGATGGTTCTGCTATCACTAAGAGATGGTCCCGCGGTGTATTAGCTAGATGGT

GAGGTAACGGCTCACCATGGCGATGATACATAGCCGAGTTGAGAGACTGATCGGCCACATTGGAACTGAG

ACACGGTCCAAACTCCTACGGGAGGCAGCAGTAGGGAATCTTCCACAATGGACGAAAGTCTGATGGAGCA

ACGCCGCGTGTGTGATGAAGGGTTTCGGCTCGTAAAACACTGTTATAAGAGAAGAATGTCGGTGAGAGTA

ACTGTTCATCGAGTGACGGTATCTTATCAGAAAGGGACGGCTAAATACGTGCCAGCAGCCGCGGTAATAC

GTATGTCCCAAGCGTTATCCGGATTTATTGGGCGTAAAGCGAGCGCAGGCGGTTATTTAAGTCTGATGTG

AAAGCCTACGGCTCAACCGTAGAATTGCATCGGAAACTGGATGACTTGAGTGCAGTAGAGGAGAGTGGAA

CTCCATGTGTAGCGGTGAAATGCGTAGATATATGGAAGAACACCAGAGGCGAAGGCGGCTCTCTGGACTG

TAACTGACGCTGAGGCTCGAAAGTGTGGGTAGCAAACAGGATTAGATACCCTGGTAGTCCACACCGTAAA

CGATGAATGCTAGTTGTTAGAGGGTTTCCGCCCTTTAGTGACGCAGCTAACGCATTAAGCATTCCGCCTG

GGGAGTACGACCGCAAGGTTGAAACTCAAAGGAATTGACGGGGGCCCGCACAAGCGGTGGAGCATGTGGT

TTAATTCGAAGCAACGCGAAGAACCTTACCAGGTCTTGACATCTTTCGCTATCTTAAGAGATTAAGAGTT

CCCTTCGGGGACGGAATGACAGGTGGTGCATGGTTGTCGTCAGCTCGTGTCGTGAGATGTTGGGTTAAGT

CCCGCAACGAGCGCAACCCTTATCATTAGTTGCCAGCATTAAGTTGGGCACTCTAATGAGACTGCCGGTG

ATAAACCGGAGGAAGGTGGGGATGACGTCAAATCATCATGCCCCTTATGACCTGGGCTACACACGTGCTA

CAATGGATGGTACAACGAGTCGCAAACTCGCGAGGGTAAGCTAATCTCTTAAAGCCATTCTCAGTTCGGA

TTGTAGTCTGCAACTCGACTACATGAAGTCGGAATCGCTAGTAATCGCGGATCAGCACGCCGCGGTGAAT

ACGTTCCCGGGCCTTGTACACACCGCCCGTCACACCA

<https://www.ncbi.nlm.nih.gov/nuccore/MH753101.1?report=fasta>

# Lactobacillus casei strain P6709 phenylalanine-tRNA ligase subunit alpha (pheS) gene, partial cds

GenBank: MH752084.1

[GenBank](https://www.ncbi.nlm.nih.gov/nuccore/MH752084.1?report=genbank) [Graphics](https://www.ncbi.nlm.nih.gov/nuccore/MH752084.1?report=graph) [PopSet](https://www.ncbi.nlm.nih.gov/popset?DbFrom=nuccore&Cmd=Link&LinkName=nuccore_popset&IdsFromResult=1607777014)

>MH752084.1 Lactobacillus casei strain P6709 phenylalanine-tRNA ligase subunit alpha (pheS) gene, partial cds

AAGAGACTTTTTATATTACCAATGAGTTACTCATGCGCTCGCAGACAAGTCCAATGCAGGCGCGGACAAT

GGAAAAGCACGACTTTACCAAAGGACCGCTGAAAATGATTAGCCCTGGGGTGGTTTATCGACGTGATGAC

GACGATGCTACTCATAGCCATCAGTTTCACCAGATGGAAGGACTCGTCATTGACAAGCATATAACCATGG

CTGATCTAAAGGGAACCTTGTTGGCCATGTGCCAACACGTGTTTGGTAAAGATCGGACAATTCGCTTGCG

GCCAAGTTATTTTCCATTTACGGAGCCATCCGTTGAAGTTGATGTTTCCTGTTTTCGTTGCGGCGGTAAA

GGTTGCCCGGTTTGCAAATATACCGGTTGGATTGAAGTGTTAGGTGCCGGCATGGTGCATCCCAATGTGC

<https://www.ncbi.nlm.nih.gov/nuccore/MH752084.1?report=fasta>

# Lactobacillus paracasei strain P6710 phenylalanine-tRNA ligase subunit alpha (pheS) gene, partial cds

GenBank: MH752080.1

[GenBank](https://www.ncbi.nlm.nih.gov/nuccore/MH752080.1?report=genbank) [Graphics](https://www.ncbi.nlm.nih.gov/nuccore/MH752080.1?report=graph) [PopSet](https://www.ncbi.nlm.nih.gov/popset?DbFrom=nuccore&Cmd=Link&LinkName=nuccore_popset&IdsFromResult=1607777006)

>MH752080.1 Lactobacillus paracasei strain P6710 phenylalanine-tRNA ligase subunit alpha (pheS) gene, partial cds

AAGAGACTTTTTACATTACCAATGAGTTACTCATGCGCTCGCAGACAAGTCCAATGCAGGCGCGGACAAT

GGAAAAGCACGACTTTACCAAAGGACCGCTGAAAATGATTAGCCCTGGGGTGGTTTATCGACGTGATGAC

GACGATGCTACTCATAGCCATCAGTTTCACCAGATGGAAGGACTCGTCATTGACAAGCATATAACCATGG

CTGATCTAAAGGGAACCTTGTTGGCCATGTGCCAACACGTTTTTGGTAAAGATCGGACAATTCGCTTGCG

GCCAAGTTATTTTCCATTTACGGAGCCATCCGTTGAAGTTGATGTTTCCTGTTTTCGTTGCGGCGGTAAA

GGTTGCCCGGTTTGCAAATATACCGGTTGGATTGAAGTGTTAGGTGCCGGCATGGTGCATCCCAATGTGC

<https://www.ncbi.nlm.nih.gov/nuccore/MH752080.1?report=fasta>

# Lactobacillus paracasei strain P6711 phenylalanine-tRNA ligase subunit alpha (pheS) gene, partial cds

GenBank: MH752081.1

[GenBank](https://www.ncbi.nlm.nih.gov/nuccore/MH752081.1?report=genbank) [Graphics](https://www.ncbi.nlm.nih.gov/nuccore/MH752081.1?report=graph) [PopSet](https://www.ncbi.nlm.nih.gov/popset?DbFrom=nuccore&Cmd=Link&LinkName=nuccore_popset&IdsFromResult=1607777008)

>MH752081.1 Lactobacillus paracasei strain P6711 phenylalanine-tRNA ligase subunit alpha (pheS) gene, partial cds

AAGAGACTTTTTATATTACCAATGAGTTACTCATGCGCTCGCAGACAAGTCCAATGCAGGCGCGGACAAT

GGAAAAGCACGACTTTACCAAAGGACCGCTGAAAATGATTAGCCCTGGGGTGGTTTATCGACGTGATGAC

GACGATGCTACTCATAGCCATCAGTTTCACCAGATGGAAGGACTCGTCATTGACAAGCATATAACCATGG

CTGATCTAAAGGGAACCTTGTTGGCCATGTGCCAACACGTGTTTGGTAAAGATCGGACAATTCGCTTGCG

GCCAAGTTATTTTCCATTTACGGAGCCATCCGTTGAAGTTGATGTTTCCTGTTTTCGTTGCGGCGGTAAA

GGTTGCCCGGTTTGCAAATATACCGGTTGGATTGAAGTGTTAGGTGCCGGCATGGTGCATCCCAATGTGC

<https://www.ncbi.nlm.nih.gov/nuccore/MH752081.1?report=fasta>

# Lactobacillus paracasei strain P6712 phenylalanine-tRNA ligase subunit alpha (pheS) gene, partial cds

GenBank: MH752082.1

[GenBank](https://www.ncbi.nlm.nih.gov/nuccore/MH752082.1?report=genbank) [Graphics](https://www.ncbi.nlm.nih.gov/nuccore/MH752082.1?report=graph) [PopSet](https://www.ncbi.nlm.nih.gov/popset?DbFrom=nuccore&Cmd=Link&LinkName=nuccore_popset&IdsFromResult=1607777010)

>MH752082.1 Lactobacillus paracasei strain P6712 phenylalanine-tRNA ligase subunit alpha (pheS) gene, partial cds

AAGAGACTTTTTACATTACCAATGAGTTACTCATGCGCTCGCAGACAAGTCCAATGCAGGCGCGGACAAT

GGAAAAGCACGACTTTACCAAAGGACCGCTGAAAATGATTAGCCCTGGGGTGGTTTATCGACGTGATGAC

GACGATGCTACTCATAGCCATCAGTTTCACCAGATGGAAGGACTCGTCATTGACAAGCATATAACCATGG

CTGATCTAAAGGGAACCTTGTTGGCCATGTGCCAACACGTTTTTGGTAAAGATCGGACAATTCGCTTGCG

GCCAAGTTATTTTCCATTTACGGAGCCATCCGTTGAAGTTGATGTTTCCTGTTTTCGTTGCGGCGGTAAA

GGTTGCCCGGTTTGCAAATATACCGGTTGGATTGAAGTGTTAGGTGCCGGCATGGTGCATCCCAATGTGC

<https://www.ncbi.nlm.nih.gov/nuccore/MH752082.1?report=fasta>

# Lactobacillus casei strain P6713 phenylalanine-tRNA ligase subunit alpha (pheS) gene, partial cds

GenBank: MH752085.1

[GenBank](https://www.ncbi.nlm.nih.gov/nuccore/MH752085.1?report=genbank) [Graphics](https://www.ncbi.nlm.nih.gov/nuccore/MH752085.1?report=graph) [PopSet](https://www.ncbi.nlm.nih.gov/popset?DbFrom=nuccore&Cmd=Link&LinkName=nuccore_popset&IdsFromResult=1607777016)

>MH752085.1 Lactobacillus casei strain P6713 phenylalanine-tRNA ligase subunit alpha (pheS) gene, partial cds

AAGAGACTTTTTACATTACCAATGAGTTACTCATGCGCTCGCAGACAAGTCCAATGCAGGCGCGGACAAT

GGAAAAGCACGACTTTACCAAAGGACCGCTGAAAATGATTAGCCCTGGGGTGGTTTATCGACGTGATGAC

GACGATGCTACTCATAGCCATCAGTTTCACCAGATGGAAGGACTCGTCATTGACAAGCATATAACCATGG

CTGATCTAAAGGGAACCTTGTTGGCCATGTGCCAACACGTTTTTGGTAAAGATCGGACAATTCGCTTGCG

GCCAAGTTATTTTCCATTTACGGAGCCATCCGTTGAAGTTGATGTTTCCTGTTTTCGTTGCGGCGGTAAA

GGTTGCCCGGTTTGCAAATATACCGGTTGGATTGAAGTGTTAGGTGCCGGCATGGTGCATCCCAATGTGC

<https://www.ncbi.nlm.nih.gov/nuccore/MH752085.1?report=fasta>

# Lactobacillus paracasei strain P6714 phenylalanine-tRNA ligase subunit alpha (pheS) gene, partial cds

GenBank: MH752083.1

[GenBank](https://www.ncbi.nlm.nih.gov/nuccore/MH752083.1?report=genbank) [Graphics](https://www.ncbi.nlm.nih.gov/nuccore/MH752083.1?report=graph) [PopSet](https://www.ncbi.nlm.nih.gov/popset?DbFrom=nuccore&Cmd=Link&LinkName=nuccore_popset&IdsFromResult=1607777012)

>MH752083.1 Lactobacillus paracasei strain P6714 phenylalanine-tRNA ligase subunit alpha (pheS) gene, partial cds

AAGAGACTTTTTACATTACCAATGAGTTACTTATGCGCTCGCAGACAAGTCCAATGCAGGCGCGGACAAT

GGAAAAGCACGACTTTACCAAAGGACCGCTGAAAATGATTAGCCCTGGGGTGGTTTATCGACGTGATGAC

GACGATGCTACTCATAGCCATCAGTTTCACCAGATGGAAGGACTCGTCATTGACAAGCATATAACCATGG

CTGATCTAAAGGGAACCTTGTTGGCCATGTGCCAACACGTTTTTGGTAAAGATCGGACAATTCGCTTGCG

GCCAAGTTATTTTCCATTTACGGAGCCATCCGTTGAAGTTGATGTTTCCTGTTTTCGTTGCGGCGGTAAA

GGTTGCCCGGTTTGCAAATATACCGGTTGGATTGAAGTGTTAGGTGCCGGCATGGTGCATCCCAACGTGT

<https://www.ncbi.nlm.nih.gov/nuccore/MH752083.1?report=fasta>

# Lactobacillus casei strain P6715 phenylalanine-tRNA ligase subunit alpha (pheS) gene, partial cds

GenBank: MH752086.1

[GenBank](https://www.ncbi.nlm.nih.gov/nuccore/MH752086.1?report=genbank) [Graphics](https://www.ncbi.nlm.nih.gov/nuccore/MH752086.1?report=graph) [PopSet](https://www.ncbi.nlm.nih.gov/popset?DbFrom=nuccore&Cmd=Link&LinkName=nuccore_popset&IdsFromResult=1607777018)

>MH752086.1 Lactobacillus casei strain P6715 phenylalanine-tRNA ligase subunit alpha (pheS) gene, partial cds

AAGAGACTTTTTACATTACCAATGAGTTACTTATGCGCTCGCAGACAAGTCCAATGCAGGCGCGGACAAT

GGAAAAGCACGACTTTACCAAAGGACCGCTGAAAATGATTAGCCCTGGGGTGGTTTATCGACGTGATGAC

GACGATGCTACTCATAGCCATCAGTTTCACCAGATGGAAGGACTCGTCATTGACAAGCATATAACCATGG

CTGATCTAAAGGGAACCTTGTTGGCCATGTGCCAACACGTTTTTGGTAAAGATCGGACAATTCGCTTGCG

GCCAAGTTATTTTCCATTTACGGAGCCATCCGTTGAAGTTGATGTTTCCTGTTTTCGTTGCGGCGGTAAA

GGTTGCCCGGTTTGCAAATATACCGGTTGGATTGAAGTGTTAGGTGCCGGCATGGTGCATCCCAACGTGT

<https://www.ncbi.nlm.nih.gov/nuccore/MH752086.1?report=fasta>

# Lactobacillus fermentum strain P6702 phenylalanine-tRNA ligase subunit alpha (pheS) gene, partial cds

GenBank: MH752076.1

[GenBank](https://www.ncbi.nlm.nih.gov/nuccore/MH752076.1?report=genbank) [Graphics](https://www.ncbi.nlm.nih.gov/nuccore/MH752076.1?report=graph) [PopSet](https://www.ncbi.nlm.nih.gov/popset?DbFrom=nuccore&Cmd=Link&LinkName=nuccore_popset&IdsFromResult=1607776998)

>MH752076.1 Lactobacillus fermentum strain P6702 phenylalanine-tRNA ligase subunit alpha (pheS) gene, partial cds

AAGACACCTTCTACGTGACCCCGTCTGTTTTGATGCGGACCCAAACGTCGCCAATGCAGGCCCGGATGCT

GGAACAACACGACTTCTCCAAGGGGCCGTTGAAGATGATCTCACCGGGGAAGGTTTACCGCCGTGACACC

GATGACGCTACCCACAGCCACCAATTCCACCAGGTTGAAGGAATCGTGGTCGGTGAACACGTCACGATGG

CCGATTTAAAGGGGACCCTAGAGGTGGTGGCCCAAAACCTGTTTGGCGACCAGCTCAAGGTGCGTCTGCG

CCCGAGTTACTTCCCATTCACGGAACCGTCCGTCGAGGCCGACATCACTTGCTTTAATTGCCTGGGGGCC

GGTTGCTCAATCTGTAAGGGGACTGGTTGGATCGAGGTGTTGGGGGCTGGAATGGTGCACCCAAACGTCT

<https://www.ncbi.nlm.nih.gov/nuccore/MH752076.1?report=fasta>

# Lactobacillus fermentum strain P6704 phenylalanine-tRNA ligase subunit alpha (pheS) gene, partial cds

GenBank: MH752077.1

[GenBank](https://www.ncbi.nlm.nih.gov/nuccore/MH752077.1?report=genbank) [Graphics](https://www.ncbi.nlm.nih.gov/nuccore/MH752077.1?report=graph) [PopSet](https://www.ncbi.nlm.nih.gov/popset?DbFrom=nuccore&Cmd=Link&LinkName=nuccore_popset&IdsFromResult=1607777000)

>MH752077.1 Lactobacillus fermentum strain P6704 phenylalanine-tRNA ligase subunit alpha (pheS) gene, partial cds

AAGACACCTTCTACGTGACCCCGTCTGTTTTGATGCGGACCCAAACGTCGCCAATGCAGGCCCGGATGCT

GGAACAACACGACTTCTCCAAGGGGCCGTTGAAGATGATCTCACCGGGGAAGGTTTACCGCCGTGACACC

GATGACGCTACCCACAGCCACCAATTCCACCAGGTTGAAGGAATCGTGGTCGGTGAACACGTCACGATGG

CCGATTTAAAGGGGACCCTAGAGGTGGTGGCCCAAAACCTGTTTGGCGACCAGCTCAAGGTGCGTCTGCG

CCCGAGTTACTTCCCATTCACGGAACCGTCCGTCGAGGCCGACATCACTTGCTTTAATTGCCTGGGGGCC

GGTTGCTCAATCTGTAAGGGGACTGGTTGGATCGAGGTGTTGGGGGCTGGAATGGTGCACCCAAACGTCT

<https://www.ncbi.nlm.nih.gov/nuccore/MH752077.1?report=fasta>

# Lactobacillus parafarraginis strain P6717 phenylalanine-tRNA ligase subunit alpha (pheS) gene, partial cds

GenBank: MH752078.1

[GenBank](https://www.ncbi.nlm.nih.gov/nuccore/MH752078.1?report=genbank) [Graphics](https://www.ncbi.nlm.nih.gov/nuccore/MH752078.1?report=graph) [PopSet](https://www.ncbi.nlm.nih.gov/popset?DbFrom=nuccore&Cmd=Link&LinkName=nuccore_popset&IdsFromResult=1607777002)

>MH752078.1 Lactobacillus parafarraginis strain P6717 phenylalanine-tRNA ligase subunit alpha (pheS) gene, partial cds

AGGATACCTTTTACTTGAGCAGTGAGTATTTGATGCGGTCACAGACCTCACCAATGCAGGCTCGAGCACT

TGAAAAACATGACTTTTCAAAGGGACCGTTGAAAATGATTTCACCAGGGATTGTGTACCGCCGCGATACT

GATGATCCGACCCATTCTCATCAATTTCATCAAGTTGAAGGCTTAGTCATTGACCGGCACATCACCATGG

CTGATTTAAAGGGCACATTGATCACAATGGCTCAAAAGATTTTTGGCGATAAATTTGATATTCGTTTACG

GCCTAGCTACTTTCCATTTACCGAACCATCAGTGGAAGTTGACGTGACCTGTTTTAATTGTATGGGTAAA

GGCTGTGACGTATGTAAACACACCGGATGGATTGAAGTCCTTGGTGCCGGAATGGTTCATCCAAACGTTC

<https://www.ncbi.nlm.nih.gov/nuccore/MH752078.1?report=fasta>

# Lactobacillus parafarraginis strain P6719 phenylalanine-tRNA ligase subunit alpha (pheS) gene, partial cds

GenBank: MH752079.1

[GenBank](https://www.ncbi.nlm.nih.gov/nuccore/MH752079.1?report=genbank) [Graphics](https://www.ncbi.nlm.nih.gov/nuccore/MH752079.1?report=graph) [PopSet](https://www.ncbi.nlm.nih.gov/popset?DbFrom=nuccore&Cmd=Link&LinkName=nuccore_popset&IdsFromResult=1607777004)

>MH752079.1 Lactobacillus parafarraginis strain P6719 phenylalanine-tRNA ligase subunit alpha (pheS) gene, partial cds

AGGATACCTTTTACTTGAGCAGTGAGTATTTGATGCGGTCACAGACCTCACCAATGCAGGCTCGAGCACT

TGAAAAACATGACTTTTCAAAGGGACCGTTGAAAATGATTTCACCAGGGATTGTGTACCGCCGCGATACT

GATGATCCGACCCATTCTCATCAATTTCATCAAGTTGAAGGCTTAGTCATTGACCGGCACATCACCATGG

CTGATTTAAAGGGCACATTGATCACAATGGCTCAAAATATTTTTGGCGATAAATTTGATATTCGTTTACG

GCCTAGCTACTTTCCATTTACCGAACCATCAGTGGAAGTTGACGTGACCTGTTTTAATTGTATGGGTAAA

GGCTGTGACGTATGTAAACACACCGGATGGATTGAAGTCCTTGGTGCCGGAATGGTTCATCCAAACGTTC

<https://www.ncbi.nlm.nih.gov/nuccore/MH752079.1?report=fasta>

# Weissella ghanensis strain P6706 phenylalanine-tRNA ligase subunit alpha (pheS) gene, partial cds

GenBank: MH752087.1

[GenBank](https://www.ncbi.nlm.nih.gov/nuccore/MH752087.1?report=genbank) [Graphics](https://www.ncbi.nlm.nih.gov/nuccore/MH752087.1?report=graph) [PopSet](https://www.ncbi.nlm.nih.gov/popset?DbFrom=nuccore&Cmd=Link&LinkName=nuccore_popset&IdsFromResult=1607777020)

>MH752087.1 Weissella ghanensis strain P6706 phenylalanine-tRNA ligase subunit alpha (pheS) gene, partial cds

AAGATACATTCTATATTACCCCTGAAATTTTAATGCGAACTCAAACATCACCAGTGCAAGCACGGACAAT

GGAAAAACATGATTTCAGTCAAGGTGCGTTGAAGATGATTTCACCTGGCCGTGTTTATCGTCGCGATACG

GATGATGCTACGCACTCACACCAATTCCATCAAGTTGAAGGTTTAGTAATTGATAAACATATCACTATGG

CTGATTTGAAAGGGACTTTATTGAAAGTTGCCCAAGAATTATTTGGTGAAAAACACCAAATACGTTTGCG

TCCTTCATACTTCCCATTTACTGAGCCGTCAGTTGAAGTCGATGTTTCATGGAATGATGTTGATGAAAAT

ACGAAACCTGAAGATATTCAATGGATTGAAGTGCTTGGTGCTGGAATGGTGCACCCAAATGTCT

<https://www.ncbi.nlm.nih.gov/nuccore/MH752087.1?report=fasta>
